# Gelatin-/Alginate-Based Hydrogel Scaffolds Reinforced with TiO_2_ Nanoparticles for Simultaneous Release of Allantoin, Caffeic Acid, and Quercetin as Multi-Target Wound Therapy Platform

**DOI:** 10.3390/pharmaceutics16030372

**Published:** 2024-03-07

**Authors:** Marija M. Babić Radić, Marija Vukomanović, Jasmina Nikodinović-Runić, Simonida Tomić

**Affiliations:** 1 University of Belgrade, Faculty of Technology and Metallurgy, Karnegijeva 4, 11000 Belgrade, Serbia; simonida@tmf.bg.ac.rs; 2 Advanced Materials Department, Jožef Stefan Institute, Jamova Cesta 39, 1000 Ljubljana, Slovenia; marija.vukomanovic@ijs.si; 3 University of Belgrade, Institute of Molecular Genetics and Genetic Engineering, Vojvode Stepe 444a, 11000 Belgrade, Serbia; jasmina.nikodinovic@imgge.bg.ac.rs

**Keywords:** hydrogel scaffolds, gelatin-/alginate-based scaffolds, sustained release, wound healing, allantoin, caffeic acid, TiO_2_ nanoparticles

## Abstract

This study proposes synthesis and evaluation of gelatin-/alginate-based hydrogel scaffolds reinforced with titanium dioxide (TiO_2_) nanoparticles which, through their combination with allantoin, quercetin, and caffeic acid, provide multi-target therapy directed on all phases of the wound healing process. These scaffolds provide the simultaneous release of bioactive agents and concurrently support cell/tissue repair through the replicated structure of a native extracellular matrix. The hydrogel scaffolds were synthesized via a crosslinking reaction using EDC as a crosslinker for gelatin. Synthesized hydrogel scaffolds and the effect of TiO_2_ on their properties were characterized by structural, mechanical, morphological, and swelling properties, and the porosity, wettability, adhesion to skin tissue, and simultaneous release features. The biocompatibility of the scaffolds was tested in vitro on fibroblasts (MRC5 cells) and in vivo (*Caenorhabditis elegans*) in a survival probe. The scaffolds revealed porous interconnected morphology, porosity of 88.33 to 96.76%, elastic modulus of 1.53 to 4.29 MPa, full hydrophilicity, favorable skin adhesivity, and biocompatibility. The simultaneous release was investigated in vitro indicating dependence on the scaffold’s composition and type of bioactive agents. The novel scaffolds designed as multi-target therapy have significant promise for improved wound healing in a beneficial and non-invasive manner.

## 1. Introduction

Wound healing is a complex and dynamic multiple-phase process for the regeneration of damaged tissue that requires coordinated interactions among dermal and epidermal cells, growth factors, cytokines, and chemokines [1]. During the wound healing process it is necessary to direct all the phases of healing including inflammation, proliferation, and tissue remodeling to achieve tissue homeostasis and integrity [2]. Any wrong action in these phases, such as discontinuities, aberrancies, or prolongation in the process, leads to a chronic state that is more vulnerable to infections and other complications. Various conventional therapies such as wound dressings, growth factor delivery, cell and gene therapy, as well as different advanced wound care technology including nanotherapeutics, bioengineered skin grafts, stem cell therapy, and 3D tissue bioprinting have been used for wound healing but they do not offer effective results for all wound types [3,4,5]. Despite the development of various wound healing approaches there is an urgent need to develop novel innovative treatment modalities for multi-target therapeutic regimens for wounds.

One of the most promising wound healing approaches is to use three-dimensional (3D) scaffolds as dermal substitutes that replicate the extracellular matrix and provide structural support for cell adhesion, migration, and proliferation [6]. Among various biomaterials, natural polymers including collagen, gelatin, chitosan, and alginate have been utilized to obtain dermal substitutes due to their favorable biological properties such as high biocompatibility, biodegradability, and ability to mimic the extracellular matrix that supports the migration and proliferation of cells during the wound healing process [7,8,9]. Collagen, gelatin, chitosan, and alginate are the best types of natural polymer for dermal scaffold design. Gelatin, obtained by the hydrolysis of collagen due to its high biocompatibility, biodegradability, higher water-binding capacity, antimicrobial activity, lower immunogenicity, and cost-effectiveness, is a suitable biomaterial for dermal tissue engineering applications [10,11,12]. Gelatin is also a hemostatic agent and contains Arg-Gly-Asp sequences in the structure which are favorable for cell adhesion [6,12,13]. Alginate, an anionic polysaccharide obtained from cell walls of brown algae, is a biocompatible, biodegradable polymer that has been used to create highly biocompatible hydrogel scaffolds favorable for wound healing applications [14]. However, weak mechanical properties limit the use of natural polymers as scaffolding biomaterials but through their mutual combination or with inorganic compounds that act as fillers such as nanoparticles of graphene oxide (GO), titanium dioxide (TiO_2_), or zinc oxide (ZnO), it is possible to provide stronger mechanical properties and adequate flexibility that allow easy handling during the wounds healing treatment [15,16].

Numerous studies in the field of dermal tissue engineering have been performed to evaluate the potential of hydrogel scaffolds for drug delivery in wound areas, and have obtained results indicating that single active agent delivery does not have a favorable effect for all wound types. From the perspective of successful wound healing treatment, the key purpose of scaffolds for drug delivery is to provide adequate therapeutic support in all three phases of dermal tissue repair [3]. Therefore, the goal of this study was to develop an innovative wound healing approach based on multi-target therapy utilizing the synergistic effect of the biocompatible scaffold and a combination of the bioactive agents with wound healing potential, namely allantoin, quercetin, and caffeic acid. Allantoin is an active natural compound that stimulates wound healing by improving local granulation, modulating the inflammatory response, and supporting the proliferation of fibroblasts, as well as collagen and ECM synthesis, leading to a more organized dermal tissue [17,18]. Caffeic acid is an active component of propolis with significant anti-inflammatory, immunomodulatory, and antioxidant activity, which is beneficial for the wound healing process due to the fact that it provides myeloperoxidase activity, inhibits lipid peroxidation, stimulates of collagen-like synthesis in fibroblast cells, and enhances re-epithelialization by decreasing oxidative stress [19,20,21,22]. Quercetin is a polyphenolic plant-derived flavonoid with strong anti-inflammatory, antioxidant, and antifibrotic activity, as well as the ability to prevent fibrosis and scars during the wound healing process [23]. The hydrogel scaffolds, through their combination with allantoin, quercetin, and caffeic acid, provide multi-target therapy to wounds directed on the inflammation, proliferation, and tissue remodeling phases by therapeutic activity of the used agents and simultaneously support growth and reparation of the cells through replicated structure of native ECM by hydrogel scaffolds.

The novel biocompatible hydrogel scaffolds were prepared via a simple crosslinking reaction using natural polymers gelatin and alginate, with varying amounts of alginate. The extra bioactivity of the hydrogel scaffolds was achieved by loading the three active agents with the ability to direct at multiple therapeutic targets during the wound healing process. Additionally, to obtain gelatin-/alginate-based scaffolds with enhanced mechanical properties, TiO_2_ nanoparticles were incorporated into the scaffolds’ composition, and the effect of TiO_2_ on the porosity, morphological, mechanical, hydrophilicity, swelling, and drug delivery behavior of the hydrogel scaffolds was investigated. The functional properties of the hydrogel scaffolds, which are important for biomedical and drug delivery engineering applications, were tested. Biological evaluation of the hydrogel scaffolds was performed through in vitro and in vivo tests by a fibroblast cell line and microworm *Caenorhabditis elegans* (*C. Elegans*). The in vitro simultaneous release study of allantoin, quercetin, and caffeic acid was monitored to evaluate the potential of hydrogel scaffolds as a multi-targeted delivery system for wound healing therapy.

## 2. Materials and Methods

### 2.1. Materials

The natural polymers gelatin (G, Type A powder, bioreagent, suitable for cell culture) and sodium alginate (A, biomedical polymer), nanoparticles of titanium (IV) oxide (TiO_2_) (rutile nanopowder, particle size < 100 nm, Mw 79.87 g/mol), crosslinker 1-ethyl-3-(3-dimethyl aminopropyl) carbodiimide hydrochloride (EDC, 98.0%) were purchased from Sigma-Aldrich, St. Louis, MO, USA. Materials used for MTT test on MRC5 cells and *Caenorhabditis elegans*: RPMI-1640 medium and supplements for cell proliferation as well as 3-(4,5-dimethylthiazol-2-yl)-2,5 diphenyltetrazolium bromide (MTT) reduction assay components, purchased from Sigma-Aldrich, St. Louis, MO, USA. Potassium hydrogen phosphates (KH_2_PO_4_ and K_2_HPO_4_, Sigma-Aldrich) were used for buffer preparations. Quercetin, caffeic acid, and allantoin were used as the bioactive agents with wound healing properties, and were purchased from Sigma-Aldrich, USA. All syntheses and experiments were performed using lab-produced, ultra-distilled water.

### 2.2. Hydrogel Scaffolds Synthesis

Hydrogel scaffolds (HS) composed of natural biomacromolecules gelatin and alginate as well as gelatin-/alginate-based scaffolds reinforced with TiO_2_ nanoparticles were synthesized by a crosslinking reaction with varying gelatin/alginate ratios (Table 1). The initial reaction mixture containing previously dissolved gelatin and sodium alginate in ultra-distilled water at 40 °C was stirred at room temperature for 1 h, then 750 µL of 1 M solution of crosslinker EDC was added and stirred for 4 min at room temperature. The reaction mixture was poured into a Petri dish and placed at −18 °C for 24 h to complete the gelation. The scaffolds were immersed in distilled water for 7 days, with daily changes of water. The hydrogels in a swollen state were placed at −80 °C and were exposed to the lyophilization process. Titanium nanoparticles as well as bioactive compounds with wound healing properties allantoin, caffeic acid, and quercetin (5% of polymer content) were loaded into the scaffolds during synthesis by adding into the initial reaction mixture.

### 2.3. Hydrogel Scaffold Characterization

#### 2.3.1. Fourier Transform Infrared Spectroscopy (FTIR)

The chemical composition of the hydrogel scaffolds was analyzed by FTIR spectra obtained by a Thermo FisherScientific Nicolet 6700 FTIR ATR taste method [16].

#### 2.3.2. Scanning Electron Microscopy (SEM)

Morphological analysis of the samples was performed through the procedure previously described [16].

### 2.4. Porosity Measurements

The porosity of the hydrogel scaffolds was calculated by the solvent replacement method using the true and bulk density of the hybrid scaffolds as well as the density obtained by the Archimedes method, where glycerol (ρ = 1.2038 g/cm^3^) was used as a wetting medium [24,25].

### 2.5. Mechanical Testing

The mechanical properties of the samples were determined by the procedure previously described [16].

### 2.6. In Vitro Swelling Study

The capacity of the samples to swell was evaluated by the procedure previously described [26,27,28].

### 2.7. Water Contact Angle Measurements

The surface hydrophilicity of the hydrogel scaffolds was analyzed by static water contact angle measurement as mentioned earlier [16].

### 2.8. Adhesiveness Test

The adhesive properties of the hydrogel scaffolds on skin tissue were evaluated by attaching swollen (in a buffer solution of pH 7.4) hydrogel scaffold to skin tissue of the moving joint with an intersection angle between 0° and 120° [29]. Adhesion of the scaffold at the finger extended at 0°, flexed to 45°, 90°, and 120°, as well as reverse attached to the palm side of a finger were photographed.

### 2.9. Biocompatibility Probes

#### 2.9.1. In Vitro Cytotoxicity Assay

The cytotoxic activities of the hydrogel scaffolds were analyzed by the method described earlier [30]. The antiproliferative activity of the hydrogel scaffolds was measured using MTT assay by the procedure described previously [16].

#### 2.9.2. In Vivo Caenorhabditis Elegans Survival Evaluation

*C. elegans* N2 (glp-4; sek-1) evaluation was performed by the procedure described previously [31,32,33].

#### 2.9.3. In Vitro Simultaneous Release Study

The hydrogel scaffolds loaded with three active agents with wound healing potential, allantoin, quercetin, and caffeic acid, were placed in a basket stirrer containing 800 mL of the release medium of phosphate buffer of pH 7.4 at 33.5 °C that simulated physiological conditions. The concentration of released allantoin, quercetin, and caffeic acid from the hydrogel scaffolds was monitored by taking the absorbance of the released media in determined time using a UV/Vis spectrophotometer (Shimadzu UV/Vis Spectrophotometer UV-1800, Kyoto, Japan) at λ_max_ values of 204 nm (allantoin), 380 nm (quercetin), and 280 nm (caffeic acid) [34,35,36].

## 3. Results and Discussion

### 3.1. Preparation of the Hydrogel Scaffolds

**Scheme 1:**
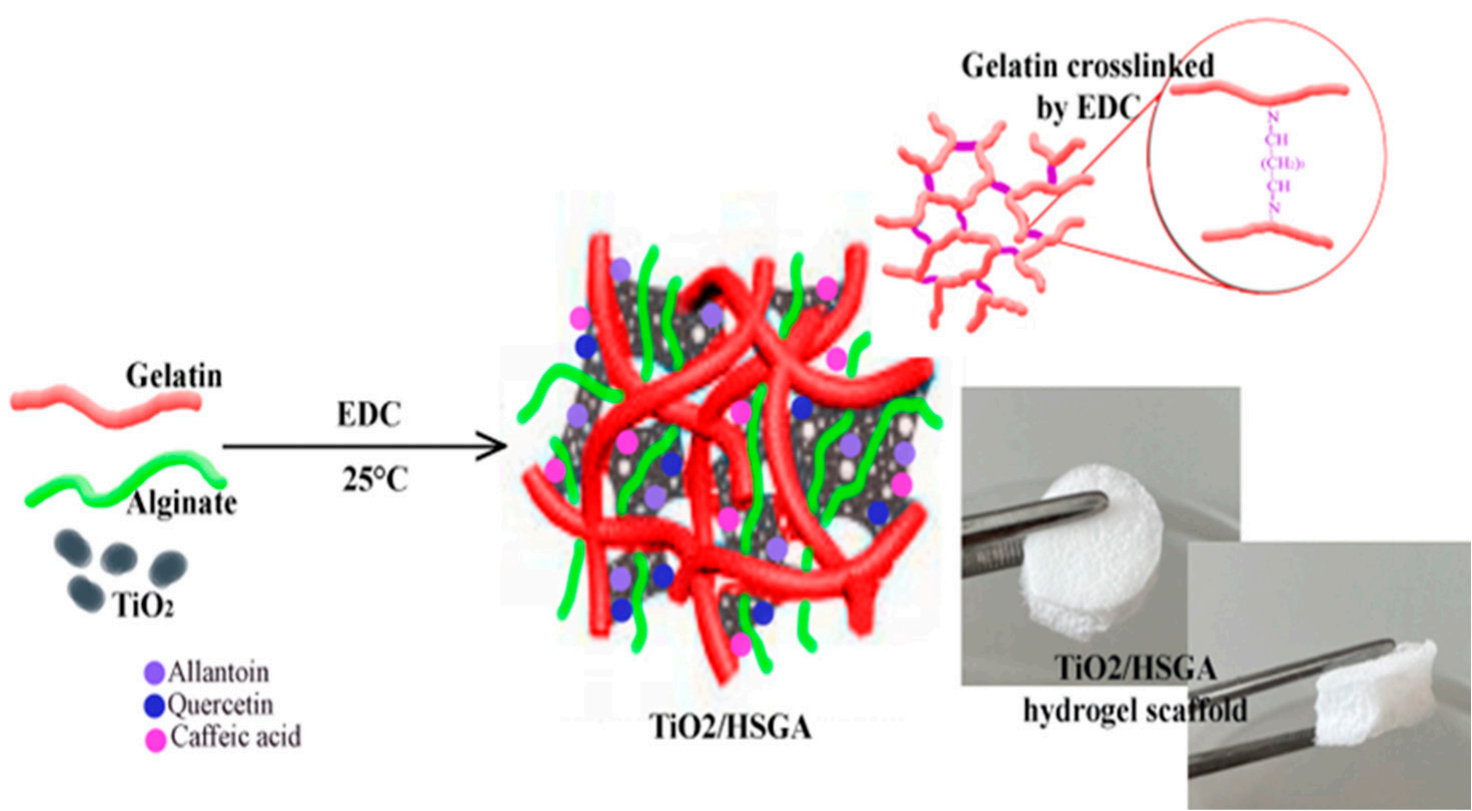
Synthesis route for the hydrogel scaffolds based on gelatin, alginate, active agents, and TiO_2_ nanoparticles obtained by a crosslinking reaction (details: photographs of the sample).

Novel hydrogel scaffolds based on polymers of natural origin, gelatin and alginate, reinforced with TiO_2_ nanoparticles and loaded with bioactive agents with wound regeneration potential including allantoin, quercetin, and caffeic acid, were synthesized by a crosslinking reaction. The highly porous structure of the hydrogel scaffolds was engineered by gelatin crosslinked with EDC, intertwined with linear alginate chains, and enriched with TiO_2_ nanoparticles (as presented in Scheme 1) to create bioactive scaffolding biomaterial for the simultaneous release of bioactive agents for wound tissue regeneration. The composition and marks of the hydrogel scaffolds are presented in Table 1.

Interpenetrating polymeric networks (IPNs) are a special kind of hydrogel where at least one polymeric network is formed in the presence of the another other. Hydrogels obtained by IPN formation possess unique performance due to the synergy of individual properties of two or more polymers. The development of IPNs is attractive due to their 3D structure providing free space for easy loading and release of active agents as well as support for cell/tissue reparation through the replicated structure of a native extracellular matrix. The novel gelatin-/alginate-based interpenetrating hydrogel networks were synthesized by crosslinking gelatin with EDC in the presence of different concentrations of alginate as a linear interpenetrant. EDC molecules provided the linking of two amino acid side chains, and introduced a stable crosslink between gelatin molecules resulting in the formation of a stable polymeric network. In the obtained IPNs, among the presence of the gelatin-EDC crosslinks, the physical interactions, mainly H-bonding, between gelatin and alginate have been formed also.

### 3.2. Structural Characteristics of the Hydrogel Scaffolds—FTIR Analysis

**Figure 1:**
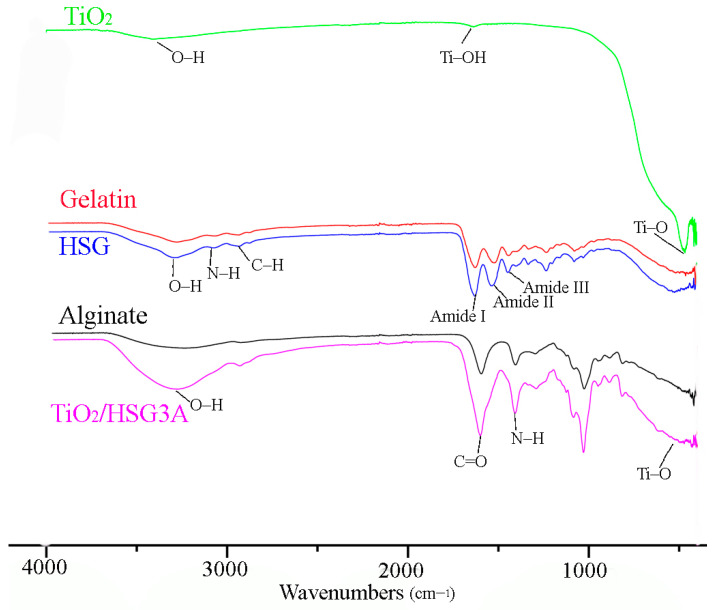
FTIR spectra of pure TiO_2_, gelatin, alginate, and gelatin-based hydrogel scaffolds HSG, and gelatin-/alginate-based hydrogel scaffold reinforced with TiO_2_ nanoparticles TiO_2_/HSG3A.

The FTIR spectra of pure gelatin, alginate, and TiO_2_, as well as of gelatin (HSG) and gelatin/alginate hydrogel scaffolds reinforced with TiO_2_ nanoparticles (TiO_2_/HSG3A) are presented in Figure 1. The FTIR spectra of both hydrogel scaffolds have the most gelatin- characteristic IR absorption bands, appearing at around 3283 cm^−1^, 1628 cm^−1^, 1539 cm^−1^, and 1441 cm^−1^, related to N–H, C=O vibrations for the amide I, N–H definition for the amide II, and amide III, respectively [16,37]. The intensity of the amide I, amide II, and amide III peaks of the HSG scaffold are higher than for pure gelatin, which might indicate that HSG contains a higher number of amide bonds due to the higher gelatin/EDC crosslinking density. Other significant absorption bands were identified in the TiO_2_/HSG3A spectrum and confirmed the presence of alginate and TiO_2_ nanoparticles in the hydrogel scaffold [38,39]. The signals at 3281 cm^−1^, 1290 cm^−1^, 1160 cm^−1^, and 1028 cm^−1^ present in the FTIR spectrum of TiO_2_/HSG3A are representative bands of alginate that are attributed to O–H stretching, C–O stretching, C–C stretching, and C–O–C stretching. The molecular compatibility among gelatin, alginate, and TiO_2_ has been studiously analyzed previously [39,40,41]. The proof of the intermolecular connections and good chemical compatibility of gelatin and alginate is the shift to the lower wavenumber IR absorption band that is related to the stretching vibration of the N-H group bonded to the O-H group, as found in the FTIR spectrum of TiO_2_/HSG3A [40,41,42]. The signals at 1623 cm^−1^ and 3404 cm^−1^ in the FTIR spectrum of pure TiO_2_ are attributed to O–H stretching. The obtained FTIR spectral changes are evidence of crosslinking of gelatin by EDC, which couples carboxyl groups with amino groups forming amide bonds as well as physical crosslinking (H-bonding) between the hydroxyl groups of alginate and the carboxyl groups of gelatin.

### 3.3. Morphology of the Hydrogel Scaffolds—SEM Analysis

**Figure 2:**
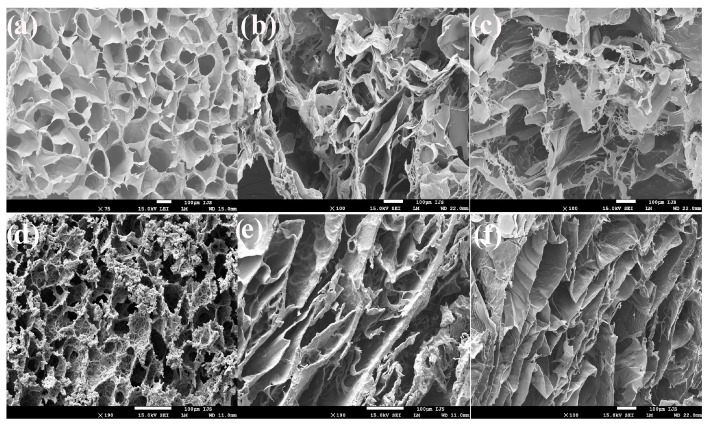
SEM micrographs of a cross-section of (**a**) HSG, (**b**) HSGA, (**c**) HG3A, (**d**) TiO_2_/HSG, (**e**) TiO_2_/HSGA, and (**f**) TiO_2_/HSG3A hydrogel scaffold.

The basic biological function of hydrogel scaffolds in tissue regeneration applications is to ensure a porous three-dimensional microstructure with interlinked pores that promotes adhesion, migration, proliferation of cells, and adequate flow of nourishment, oxygen, and active agents needed for successful reparation of damaged tissue. Therefore, the morphology of the synthesized hydrogel scaffolds was observed by SEM. The obtained SEM micrographs (Figure 2) of the hydrogel scaffolds indicate a highly porous interconnected morphology favorable for tissue engineering applications. The pores in the gelatin-based scaffolds are small and regular with homogeneous thin walls (Figure 2a). The incorporation of alginate into the scaffolds’ composition modifies the pattern of morphology and increases porosity. The pores in scaffolds containing alginate are large and irregular with thicker walls and a disorganized structure (Figure 2b,c). Reinforcing the hydrogel scaffolds with TiO_2_ nanoparticles also leads to a change pattern of morphology and decreases porosity. The incorporation of TiO_2_ nanoparticles into the gelatin-based scaffold reduces the size of pores and increases the thickness of the walls (Figure 2d). Layered morphology with elongated pores reduced in size and thick walls was observed for the scaffolds containing alginate reinforced with TiO_2_ nanoparticles (Figure 2e,f). The reduction of porosity of the hydrogel scaffolds reinforced with TiO_2_ nanoparticles can be related to the fact that TiO_2_ nanoparticles are excellent inorganic fillers with potential to enhance the mechanical performance of the biomaterials. Based on SEM results, the morphology of the scaffolds can be simply tuned following the requirements of their final applications by the variation of the scaffold composition.

### 3.4. Porosity of the Hydrogel Scaffolds

The potential of biomaterial to serve as an efficient scaffold for tissue regeneration is determined by the porosity—the free space of its inner structure that affects the biological and mechanical properties of scaffolding biomaterial. Inadequate porosity reduces the vital functions of cells inside the scaffold. A scaffold porosity above 50% is considered to be suitable for successful cell populations [43,44,45]. The obtained results of the porosity measurements for the scaffolds are summarized in Table 2, indicating dependence on the scaffolds’ composition. The porosity values of the gelatin-/alginate-based scaffolds are from 92.42% to 96.74% which is favorable for biomedical/tissue engineering applications. For the scaffolds reinforced with TiO_2_ nanoparticles, the porosity values are slightly reduced probably due to TiO_2_ nanoparticles filling the free space in the polymeric network. However, the porosity of the gelatin-/alginate-based scaffolds reinforced with TiO_2_ nanoparticles is from 88.33% to 92.92% which is also suitable for biomedical and tissue engineering applications.

### 3.5. Mechanical Properties of the Hydrogel Scaffolds

The structural functionality and durability of scaffolding biomaterial are determined by their mechanical properties, which are crucial requirements for their tissue engineering applications. The mechanical properties of gelatin, gelatin/alginate, and hydrogel scaffolds reinforced with TiO_2_ nanoparticles were analyzed by Young’s modulus values (E) (Table 2).

Mechanical analysis results revealed a dependence of value of Young’s moduli on the hydrogel scaffold’s composition as well as on the degree of crosslinking. The highest value of Young’s modulus was obtained with gelatin-based scaffolds reinforced with TiO_2_ nanoparticles related to gelatin chemically crosslinked with EDC, and physically crosslinked with TiO_2_ nanoparticles, as well as filled polymeric network with TiO_2_ nanoparticles. The incorporation of alginate into the composition of the gelatin-based scaffolds decreases the value of Young’s modulus, indicating that chemical crosslinking affects the mechanical properties. More precisely, a larger amount of chemically crosslinked gelatin in the hydrogel scaffolds’ composition improves their mechanical properties. On the other side, the incorporation of TiO_2_ nanoparticles into the scaffolds improves their mechanical properties. For example, the value of the Young’s modulus of TiO_2_/HSG3A is 2.7 times higher than that of the same scaffold without TiO_2_ nanoparticles. The changes in the elastic modulus caused by the incorporation of TiO_2_ nanoparticles in the scaffold’s composition could be related to forming additional physical crosslinks between hydroxyl groups of TiO_2_ and hydroxyl groups of alginate; also, TiO_2_ nanoparticles are excellent fillers that can improve the mechanical properties of the materials. From obtained mechanical analysis results, it is clear that the mechanical properties of the hydrogel scaffolds could be engineered by adjusting the selection of chemical components and the degree of crosslinking.

### 3.6. Swelling Properties of the Hydrogel Scaffolds

The ability of the scaffolding biomaterials to absorb and maintain large amounts of biological fluids is a crucial property necessary for functional tissue development [46]. The hydrogel scaffolds’ ability to swell was tested in a buffer medium of pH 7.40 at 33.5 °C that simulated biological conditions. The results are summarized in Table 2 as values of the equilibrium degree of swelling (*q_e_*). The dependence of swelling capacity on the scaffolds’ composition was observed and values of *q_e_* are from 8.99 to 25.38 for the gelatin-/alginate-based scaffolds and from 7.10 to 15.16 for the gelatin-/alginate-based scaffolds reinforced with TiO_2_ nanoparticles. The incorporation of alginate in the scaffolds’ composition improved swelling ability while TiO_2_ nanoparticles slightly reduced values of *q_e_* due to their acting as a filler that occupies free space in the polymeric network, hindering the relaxation of polymeric chains and the diffusion of fluid into the material. The hydrogel scaffolds containing higher amounts of alginate show a higher value of *q_e_* relating to the reduced degree of crosslinking due to the lower content of chemically crosslinked gelatin. Based on obtained results, it is clear that swelling capacity can be tuned in a wide range (from 7 to 25) by modifying the chemical composition of the scaffolds, which increases their potential for biomedical tissue engineering applications.

### 3.7. Hydrophilicity of the Hydrogel Scaffolds

The potential of scaffolding biomaterial to serve as an effective tissue substituent that promotes cell adhesion and proliferation, and reparation of damaged tissue depends also on surface hydrophilicity because the cells realize the first contact with the biomaterial through the surface of the biomaterial. The hydrophilicity of the material’s surfaces was determined by the contact angle measurements (hydrophilic surface < 90°, hydrophobic surface > 90°) and the results performed at 0 s are presented in Table 2. The results revealed fully hydrophilic surfaces of HSG3A, TiO_2_/HSG, TiO_2_/HSGA, and TiO_2_/HSG3A—the drop of water in contact with the scaffold’s surface immediately disappeared. HSG and HSGA showed slower disappearance of the water drop, but the obtained values of the contact angle are less than 90°, classifying their surfaces as hydrophilic also.

### 3.8. Adhesion Properties to the Skin Tissue of the Hydrogel Scaffolds

**Figure 3:**
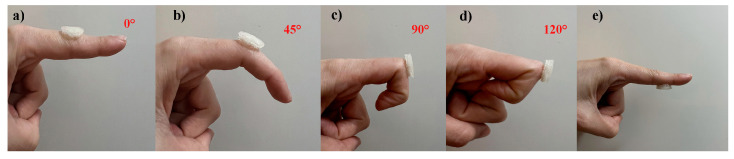
Adhesion of gelatin-/alginate-based hydrogel scaffold reinforced with TiO_2_ nanoparticles: photographs of (**a**) finger extended at 0° with the scaffold attached, (**b**) finger flexed to 45° with the scaffold attached, (**c**) finger flexed to 90° with the scaffold attached, (**d**) finger flexed to 120° with the scaffold attached, (**e**) the scaffold reverse attached to the palm side of finger.

The disadvantage of conventional hydrogel dressings is that they have to be fixed with gauze or other additional fixation systems which cannot provide enough drainage of the wound and reduce the efficiency of wound healing. The adhesive properties of the hydrogel dressings are important for successful wound healing because firmly adhering hydrogels prevent the leakage of exudate or gas from the wound as well as bacterial infections. Numerous scientific strategies have been developed to improve the tissue adhesion properties of the hydrogels such as enhancing the interfacial force with the tissue by mussel-inspired hydrogels via a Michael-type reaction of catechol/quinone groups with amino/thiol groups on the protein or oxidized dextran hydrogel via a Schiff base reaction between aldehyde groups on hydrogels and amino groups from tissue [47,48]. The adhesive properties of the hydrogel scaffolds were tested by attaching the scaffold to skin tissue on a moving joint, and the obtained photographs are shown in Figure 3. As can be seen from Figure 3, stable attachment of the scaffold on skin tissue with an intersection angle from 0° to 120° was achieved. The scaffold that was reverse attached to skin tissue also provided stable adhesion (Figure 3e). It was found that the hydrogen bond and electrostatic interactions between biomaterial and skin tissue contribute to their adhesive properties [49]. Gelatin can provide skin tissue adhesion due to a large number of hydrogen bond-forming sites such as carboxyl, amine, and hydroxyl groups. Alginate and TiO_2_ nanoparticles can form H-bonds via a hydroxyl group. The obtained skin tissue adhesion properties of the hydrogel scaffolds correspond to hydrogen bond interaction between H-bond-forming groups of gelatin, alginate, and TiO_2_ nanoparticles as well as interactions between amino groups of gelatin and oppositely charged phospholipids in the biomembranes of skin tissue [50].

### 3.9. Biocompatibility Assays of the Hydrogel Scaffolds

**Figure 4:**
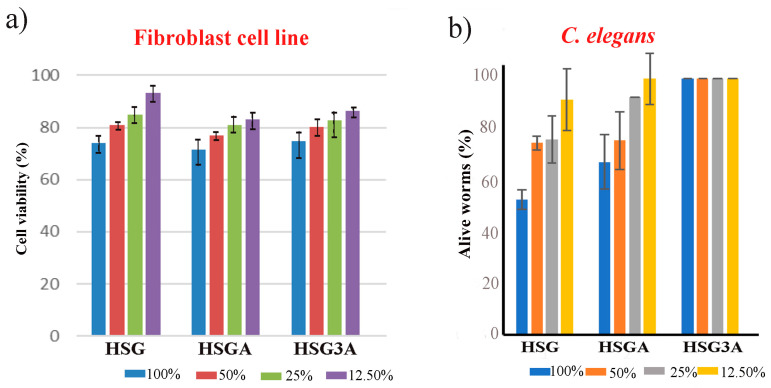
(**a**) MRC5 cell viability in the presence of 12.50%, 25%, 50%, and 100% *v*/*v* of extracts of HSG, HSGA, and HSG3A scaffolds. (**b**) Nematodotoxicity of 12.50%, 25%, 50%, and 100% *v*/*v* of extracts of HSG, HSGA, and HSG3A scaffolds.

Biocompatibility is a decisive criterion for the use of scaffolding materials in biomedical and tissue engineering applications. The biocompatibility of the synthesized hydrogel scaffolds was tested by MTT assay as well as in vivo by *C. elegans* survival assay, and the obtained results are shown in Figure 4. The in vitro cell viability of the hydrogel scaffolds was tested on an MRC5 cell line treated with different concentrations of the hydrogel scaffold extracts. The obtained results (Figure 4a) show biologically accepted values of cell viability treated with the hydrogel scaffolds’ extracts, indicating the in vitro cytocompatibility of the hydrogel scaffolds.

The nematode *C. elegans* takes primacy over mammalian animals for use in predictive toxicology and drug discovery because of its very short generation time, low-cost cultivation, and high similarity of tissues and genes with humans [51,52]. *C. elegans* was used for in vivo assay to evaluate the biomedical potential of the materials, and the results are shown in Figure 4b. The gelatin-/alginate-based hydrogel scaffold with a higher amount of alginate HSG3A was safe at all applied concentrations. The scaffolds HSG and HSGA were moderately nematodotoxic at concentrations of 100%, 50%, and 25% and slightly nematodotoxic at a concentration of 12.5%. The incorporation of alginate into the scaffold composition increased the favorable in vivo response. Based on the results of the biocompatibility assay, the synthesized HSG3A scaffold with a higher amount of alginate shows special potential to serve as an efficient biomaterial for tissue engineering applications.

### 3.10. In Vitro Simultaneous Release Properties of the Hydrogel Scaffolds

The process of loading the active agents into the hydrogel scaffolds used in this study is simple and has some advantages compared to conventional loading by absorption. The loading of the active agents was achieved by mixing a precise amount of the active agents with the pre-hydrogel reaction mixture (initial mixture), which decreased the loading process time and as a result, gave a formulation with a precise dose of the active agents. The hydrogel scaffolds were loaded with the same concentration (5% *w*/*w* respect to gelatin-/alginate-based scaffold) of the active agents allantoin, caffeic acid, and quercetin.

**Figure 5:**
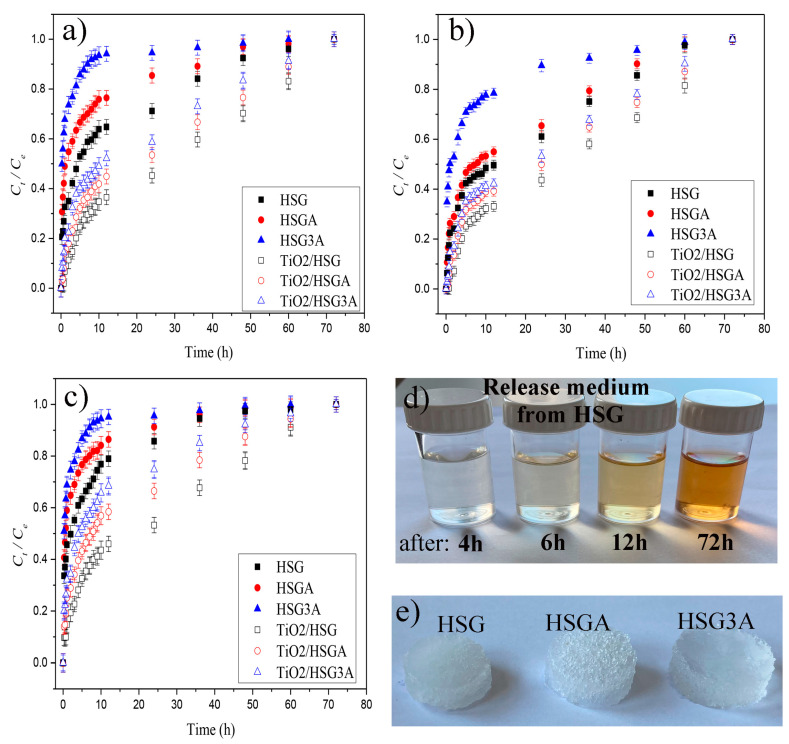
Release profiles of (**a**) caffeic acid, (**b**) allantoin, and (**c**) quercetin from the gelatin-/alginate-based hydrogel scaffolds and gelatin-/alginate-based hydrogel scaffolds reinforced with TiO_2_ nanoparticles; (**d**) photographs of the release medium after 4 h, 6 h, 12 h, and 72 h of release from the HSG hydrogel scaffold; (**e**) photographs of HSG, HSGA, and HSG3A hydrogel scaffolds after release of the bioactive agents.

The potential of the hydrogel scaffolds as a simultaneous release platform for multi-target therapy in the wound healing process was evaluated by an in vitro release study of the active agents in simulated physiological conditions (pH 7.4, at 33.5 °C). The obtained simultaneous active agents’ release profiles from the hydrogel scaffolds are presented in Figure 5.

The release results revealed a dependence of release behavior on the hydrogel scaffold’s composition as well as on the type of bioactive agent. The gelatin-/alginate-based scaffolds provided a rapid release of the active agents into the release media during the first 6 h of the release while gelatin/alginate scaffolds reinforced with TiO_2_ nanoparticles provided slightly slower release during the same release period. As shown in Table 3, after the first 6 h of release, 44% of allantoin, 56% of caffeic acid, and 66% of quercetin had been released from the gelatin-based scaffold while 27% of allantoin, 28% of caffeic acid. and 36% of the quercetin had been released from the gelatin-based scaffold reinforced with TiO_2_ nanoparticles. The incorporation of alginate into the composition of the gelatin-based scaffolds provided faster release of the active agents compared to the gelatin-based scaffolds. For example, 49% of allantoin, 70% of caffeic acid and 79% of quercetin were released from the gelatin-/alginate-based scaffold with equal amounts of both natural polymers, while 73% of allantoin, 88% of caffeic acid, and 89% of quercetin were released from the gelatin/alginate scaffold containing three times as much alginate as gelatin. After this initial fast release phase, all the scaffolds showed a slower bioactive agents release (Figure 5) until 3 days. The percentages of release rate of bioactive agents after 6 and 12 h of release are depicted in Table 3 for each scaffold. The initial rapid release phase (during the first 6 h of release) (Table 4) was followed by a slower release of the active agents (Table 5).

As shown in Figure 5, the release patterns of the bioactive agents from the hydrogel scaffolds were dependent on the scaffold’s composition as well as on the type of bioactive agent. The incorporation of alginate and TiO_2_ nanoparticles into the composition of the gelatin-based scaffold showed the opposite effect on the release properties—alginate increased while TiO_2_ nanoparticles decreased the release rate of the bioactive agents. This phenomenon can be explained by the high hydrophilicity of alginate, which can absorb large quantities of water and biological fluids, swell, and accelerate the release rate of the bioactive agents. In contrast, TiO_2_ nanoparticles act as a filler that occupies free polymeric network space and hinders the relaxation of the polymer chains and diffusion of the fluid and active agents. The gelatin-based hydrogel scaffold reinforced with TiO_2_ nanoparticles showed slower release compared to the gelatin-based scaffold due to two reasons—TiO_2_ occupies the free space in the polymeric network, hindering diffusion of the active agents, and gelatin can form an ionic interaction with TiO_2_ nanoparticles at pH 7.4 that act as additional crosslinks, reducing the release of the active agents.

The release patterns of the bioactive agents from the hydrogel scaffolds were also dependent on the type of bioactive agent. As can be seen from Figure 5, the hydrogel scaffolds provided a higher release rate of quercetin, a medium release of caffeic acid, and a slower release of allantoin. The properties of a bioactive agent, such as polarity, functional groups in its structure, and ability to interact with free functional groups in the polymeric network to form ionic interaction or hydrogen bonds, determined the release patterns from the scaffolds.

## 4. Conclusions

The novel biocompatible hydrogel scaffolds were prepared by a simple crosslinking reaction using natural polymers gelatin and alginate, with varying amounts of alginate. The extra bioactivity of the hydrogel scaffolds was achieved by loading the three active agents with the ability to direct at multiple therapeutic targets in wounds during the wound healing process. To obtain gelatin-/alginate-based scaffolds with enhanced mechanical properties, TiO_2_ nanoparticles were incorporated into the scaffolds’ composition and the effect of TiO_2_ on their properties was investigated. The physicochemical testing of the hydrogel scaffolds indicated that variation in the scaffolds’ composition permits tailoring of all properties important for drug delivery and biomedical engineering applications. The highly porous morphology with interconnected pores favorable for tissue engineering applications, tunable porosity in the range of 88.33 to 96.74%, favorable swelling capacity, adhesion to skin tissue, and biocompatibility of the hydrogel scaffolds were obtained. Successful simultaneous release of allantoin, quercetin, and caffeic acid was achieved by all hydrogel scaffolds, indicating that the release pattern could be tuned by altering the scaffolds’ chemical composition. The release data showed that the scaffolds provide controlled simultaneous release for 3 days, suggesting high potential of the hydrogel scaffolds for multi-target therapy in the wound healing process.

## Figures and Tables

**Table 1:** The scaffolds’ chemical composition and labels.

Sample	Gelatin (g)	Sodium Alginate (g)	Allantoin (g)	Quercetin (g)	Caffeic Acid (g)	TiO_2_ (g)
HSG	1.2	0	0.06	0.06	0.06	0
HSGA	0.6	0.6	0.06	0.06	0.06	0
HSG3A	0.3	0.9	0.06	0.06	0.06	0
TiO_2_/HSG	1.2	0	0.06	0.06	006	0.06
TiO_2_/HSGA	0.6	0.6	0.06	0.06	0.06	0.06
TiO_2_/HSG3A	0.3	0.9	0.06	0.06	006	0.06

**Table 2:** The results of the porosity (%), Young’s modulus (MPa), contact angle, and equilibrium degree of swelling (*q_e_*) of the hydrogel scaffolds.

Sample	Porosity (%)	Young’s Modulus (MPa)	Contact Angle	Equilibrium Degree of Swelling (*q_e_*)
HSG	92.42 ± 4.3	3.52 ± 0.16	77.55°	8.99 ± 0.5
HSGA	95.52 ± 3.9	2.46 ± 0.12	43.70°	15.66 ± 0.7
HSG3A	96.74 ± 4.0	1.53 ± 0.08	0°	25.38 ± 1.3
TiO_2_/HSG	88.33 ± 3.2	4.29 ± 0.21	0°	7.10 ± 0.4
TiO_2_/HSGA	90.10 ± 3.3	3.34 ± 0.16	0°	12.48 ± 0.5
TiO_2_/HSG3A	92.92 ± 3.5	4.24 ± 0.21	0°	15.16 ± 0.6

**Table 3:** The percentage of release rate after 6 and 12 h of bioactive agent release from the hydrogel scaffolds.

Hydrogel Scaffold	Released % of Allantoin		Released % of Caffeic Acid		Released % of Quercetin	
	6 h	12 h	6 h	12 h	6 h	12 h
HSG	44 ± 2.0	50 ± 1.2	56 ± 2.1	65 ± 1.5	66 ± 2.2	79 ± 1.6
HSGA	49 ± 1.8	55 ± 1.2	70 ± 2.0	76 ± 1.2	79 ± 2.3	86 ± 1.9
HSG3A	73 ± 2.1	78 ± 1.5	88 ± 1.8	94 ± 1.6	89 ± 2.7	95 ± 1.8
TiO_2_/HSG	27 ± 1.9	33 ± 1.8	28 ± 2.2	36 ± 1.4	36 ± 2.4	46 ± 1.9
TiO_2_/HSGA	34 ± 1.7	39 ± 1.5	35 ± 2.1	45 ± 1.5	46 ± 2.6	58 ± 1.5
TiO_2_/HSG3A	37 ± 2.0	42 ± 1.5	42 ± 2.0	52 ± 1.6	56 ± 2.4	68 ± 1.8

**Table 4:** The release data obtained during the initial period of bioactive agent release from the scaffolds.

	Initial Release Period					
Hydrogel Scaffold	Allantoin		Caffeic Acid		Quercetin	
	mg/6 h	mg/h	mg/6 h	mg/h	mg/6 h	mg/h
HSG	26.4 ± 1.20	4.4 ± 0.20	33.6 ± 1.26	5.6 ± 0.21	39.6 ± 1.32	6.6 ± 0.22
HSGA	29.4 ± 1.08	4.9 ± 0.18	42 ± 1.20	7 ± 0.20	47.4 ± 1.38	7.9 ± 0.23
HSG3A	43.8 ± 1.26	7.3 ± 0.21	52.8 ± 1.08	8.8 ± 0.18	53.4 ± 1.62	8.9 ± 0.27
TiO_2_/HSG	16.2 ± 1.14	2.7 ± 0.19	16.8 ± 1.32	2.8 ± 0.22	21.6 ± 1.44	3.6 ± 0.24
TiO_2_/HSGA	20.4 ± 1.02	3.4 ± 0.17	21 ± 1.26	3.5 ± 0.21	27.6 ± 1.56	4.6 ± 0.26
TiO_2_/HSG3A	22.2 ± 1.20	3.7 ± 0.20	25.2 ± 1.20	4.2 ± 0.20	33.6 ± 1.44	5.6 ± 0.24

**Table 5:** The release data obtained during the slow period of bioactive agent release from the scaffolds.

Slow Release Period						
Hydrogel Scaffold	Allantoin		Caffeic Acid		Quercetin	
	mg/6 h	mg/h	mg/6 h	mg/h	mg/6 h	mg/h
HSG	33.6 ± 1.08	0.51 ± 0.18	26.4 ± 1.08	0.4 ± 0.18	20.4 ± 1.44	0.31 ± 0.24
HSGA	30.6 ± 1.26	0.46 ± 0.21	18.0 ± 1.26	0.27 ± 0.21	12.6 ± 132	0.19 ± 0.22
HSG3A	16.2 ± 1.32	0.25 ± 0.22	7.20 ± 1.38	0.11 ± 0.23	6.6 ± 1.26	0.1 ± 0.21
TiO_2_/HSG	43.8 ± 1.44	0.66 ± 0.24	43.2 ± 1.26	0.65 ± 0.21	38.4 ± 1.08	0.58 ± 0.18
TiO_2_/HSGA	39.6 ± 1.26	0.60 ± 0.21	39 ± 1.14	0.59 ± 0.19	32.4 ± 1.14	0.49 ± 0.19
TiO_2_/HSG3A	37.8 ± 1.14	0.57 ± 0.19	34.8 ± 1.20	0.52 ± 0.20	26.4 ± 1.32	0.4 ± 0.22

## Data Availability

Data are contained within the article.
